# Improving HIV medication adherence among forced migrants living with HIV: a qualitative study of refugees and asylum seekers in Malaysia

**DOI:** 10.1186/s13031-022-00482-w

**Published:** 2022-09-15

**Authors:** Dasha Reddy, Nicole S. Berry

**Affiliations:** Faculty of Health Sciences, Simon Fraser University, 8888 University Dr., Burnaby, BC V5A 1S6 Canada

**Keywords:** HIV, Acquired immunodeficiency syndrome, Medication adherence, Displaced persons, Refugees, Asylum seekers, Malaysia

## Abstract

**Introduction:**

Adherence to medication regimens is essential to decrease morbidity/mortality and increase life expectancy among HIV positive persons on Highly Active Anti-retroviral Therapy (HAART). This study was born in response to the absence of information regarding access and adherence to HAART among refugee and asylum seekers in urban, irregular, transit country settings.

**Objectives:**

The purpose was to understand the barriers and facilitators to HIV medication adherence among refugees and asylum seekers living with HIV and to generate novel recommendations to facilitate adherence.

**Methods:**

Individual in-depth interviews were conducted with 34 refugees and asylum seeks to explore their lived experiences. Interviews were structured around the social ecological model to capture influences of multiple levels. Thematic analysis was conducted on transcripts.

**Results:**

Stigma, lack of knowledge and language barriers were among the main barriers noted by refugees and asylum-seekers in relation to HIV medication adherence, whereas interpersonal relationships, improved health, and strong patient-physician relationships were seen as facilitators. Participants noted their desire for community-support groups, education, and increased use of interpreters in order to combat some of the social barriers preventing full HIV medication adherence.

**Conclusion:**

A regular status shapes participants’ adherence to HIV medications. Group-based interventions to support refugees are needed.

## Introduction

The global refugee crisis is growing. According to the United Nations High Commissioner for Refugees (UNHCR), an estimated 79.5 million people worldwide have been forced to flee their homes, constituting unprecedented high levels of displacement (United Nations High Commissioner for Refugees [UNHCR] [42]). The 1951 Refugee Convention provides an international legal framework that defines the rights of displaced persons as well as the obligations of States to protect them [38, 45]. The Convention recognizes two types of persons of concern (POC): refugees,and asylum-seekers. Refugees are individuals who have gone through the process of refugee status determination by either the government or UNHCR and who have been deemed in need of international protection as per the Convention, while asylum-seekers have yet to go through refugee status determination. Refugees status protects displaced persons from being expelled into situations where their life or freedoms are at risk [43, 44].

POC’s lives are marked by insecurity on multiple levels and these insecurities influence adherence to Human Immunodeficiency Virus (HIV) medication. O’Laughlin et al. [29] found that drug stock-outs and shortages, distance to clinics, and violence and unrest were among the many challenges present within a refugee settlement camp in Uganda. Mendelsohn et al. [24] found comparable results in a settlement camp in Kenya where in addition to pharmacy stock-outs, disrupted continuity of care made it difficult for POCs to access treatment. However, participants in Mendelsohn et al.’s study also pointed to less tangible medical barriers to adherence, and these included food insecurity and emotional insecurity. Likewise, Mendelsohn et al., found that economic insecurity and fear of arrest (both the results of “illegal” or “irregular” status) contributed to lack of adherence.

Scholars have analyzed the influence of available supports within refugee communities as contributing to HIV medication adherence. This literature has been characterized by breaking down ‘support’ into standardized categories (e.g. instrumental support,^1^ informational support, etc.) to allow specific evaluation of each support type. Informational support was found to enable refugees to obtain HIV tests and access care, while emotional and instrumental support helped them cope with their diagnosis and adhere to HIV treatment [31]. Wong et al. [52] study of social support and adherence to HIV medication among migrants in Toronto, Canada further sustained the correlation between support types and medication adherence. Winston et al. [51], which looked at resettled refugee populations in the United States, additionally found that case management support may be connected to medication adherence improvements. Inquiry into the relationship between support and adherence has, therefore, been robust in that use of standardized terminology has enabled easy comparisons across studies. However, our understandings have also been limited as reliance on standardized terminology has precluded a more participatory, open approach of asking refugees themselves what they felt was needed to improve adherence to medication.

As mentioned previously, adherence to ARTs can be complicated and establishing a good relationship between a physician and patient is important. Studies show, however, that refugee patients are frequently challenged to establish and maintain such good relationships. For example, Garang et al. [12] showed that poor relationships between internally displaced persons (IDPs) and physicians in Uganda hindered information sharing, which led to poor understanding among IDPs of dosing regimens as well as the importance of taking medication, ultimately negatively impacting adherence [12].

Language differences have also been found to be a significant barrier to good physician–patient communication and relationships [32], and because refugee patients often speak a different language than their physicians, language barriers can present a significant hinderance to HIV medication adherence. Chen et al. [7] showed language barriers restricted health-seeking behaviours related to HIV adherence for immigrants, refugees and non-status folk living with HIV/AIDS in Toronto, Canada. While interpreters were noted as essential to decreasing language barriers and increasing understandability, many physicians expressed concerns over interpreters not adequately translating medical terminology or being ill-informed about HIV/AIDS. Nonetheless, Mendelsohn et al. [25] showed that language barriers for HIV positive refugees could be overcome with the effective use of interpreters and support counsellors that were directly recruited from refugee communities.

Once barriers and facilitators are elucidated, interventions aimed at enhancing HIV medication adherence can be implemented. As of now, few studies have made suggestions for or evaluated interventions to improve HIV medication adherence among refugee populations. Rouhani et al. [31] findings on the important effects of instrumental support, including helping patients pick up medication, remember medication schedules and alleviate daily tasks so that they could attend medical appointments, suggest that interventions that capitalise on individual and community networks to develop instrumental support may facilitate treatment adherence. Additionally, O’Laughlin et al. [29] suggested interventions involving freedom of movement for HIV positive refugees. Freedom of movement, defined by the Convention as the right to move freely and safely and to choose one’s residence, is hypothesized to improve refugee access to care as they seek work in different areas of their new environment [29]. From qualitative data analysing HIV medication adherence in other populations, peer support and education are suggested as the primary interventions. Peer support is hypothesized to help overcome the barrier of far distances to clinics by creating groups that rotate to help each other pick up medication [19]. Education is a suggested intervention by a Médicins Sans Frontiéres (MSF) HIV program in the Democratic Republic of Congo, which aims to spread knowledge on the importance of medication adherence [10].

In sum, adherence to HIV medication among refugee and asylum-seeker populations is a topic that has been infrequently studied. An overview of the literature shows a dominance of quantitative approaches that analyse refugees living in settlements or resettled within countries that are party to the 1951 Refugee Convention [24, 29, 31]. Given the variation among refugee/asylum-seeker backgrounds, transit circumstances (e.g., housed in refugee camps or living among a general population), and health related experiences, it is difficult to assume knowledge about challenges to adherence or generalise challenges from one setting to another. Adherence is an issue that intersects various facets of POC lives, including individual, interpersonal, environmental and community-orientation. Therefore, we need studies that reflect the variety of situations in which POC find themselves. The purpose of this study is to build off the existing literature by qualitatively exploring HIV medication adherence among refugee/asylum-seeker populations living with irregular status in urban contexts with the goal of suggesting possible interventions.

## Methods

### Setting

**Fig. 1:**
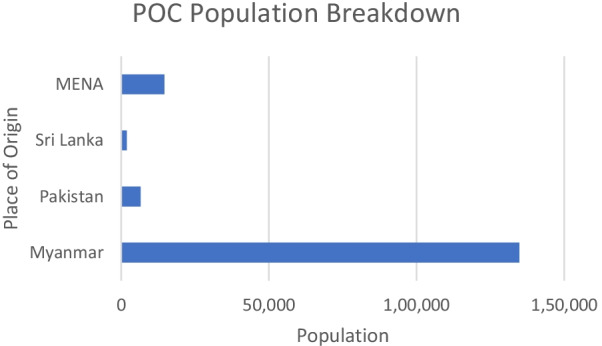
Refugee population breakdown by place of origin. MENA: Middle East & North Africa

There are currently 179,390 refugees and asylum-seekers registered with UNHCR in Malaysia, nearly double that of 2010 [41, 46]. With various countries of origin such as Pakistan, Sri Lanka, Yemen, Somalia, Syria, and Afghanistan, the majority of displaced (~ 85%) persons residing in Malaysia originally come from Myanmar (UNHCR [47]. The Rohingya population has recently become the largest proportion of those registered with UNHCR given the latest conflict arising in Rakhine State, Myanmar (Fig. 1) (UNHCR [47]. In terms of an age distribution, approximately one quarter of those registered are children under the age of 18—a slight underrepresentation compared to the global figures (UNHCR [47].

In the Malaysian context, UNHCR is the dominant body safeguarding refugee and asylum-seeker wellbeing (UNHCR Malaysia [48]), as Malaysia is not among the 145 States that have signed and ratified the 1951 Refugee Convention [45]. Therefore, Malaysia has no internationally grounded legal framework in place to protect, regulate, or monitor refugees and asylum-seekers that reside within the borders [3]. Failure to be party to the Convention has resulted in no formal distinction made between refugees/asylum-seekers and undocumented migrants—all of whom the government considers are illegally residing [35]. Indeed, illegal or irregular status, further exposes these vulnerable populations to exploitation, insecurity and uncertainty,they must find informal ways to work, gain education, and access health services, among many other daily challenges [28, 38, 39].

**Fig. 2:**
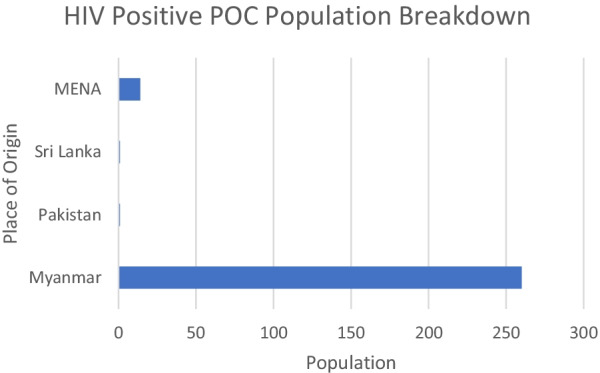
HIV positive POC breakdown by place of origin

**Fig. 3:**
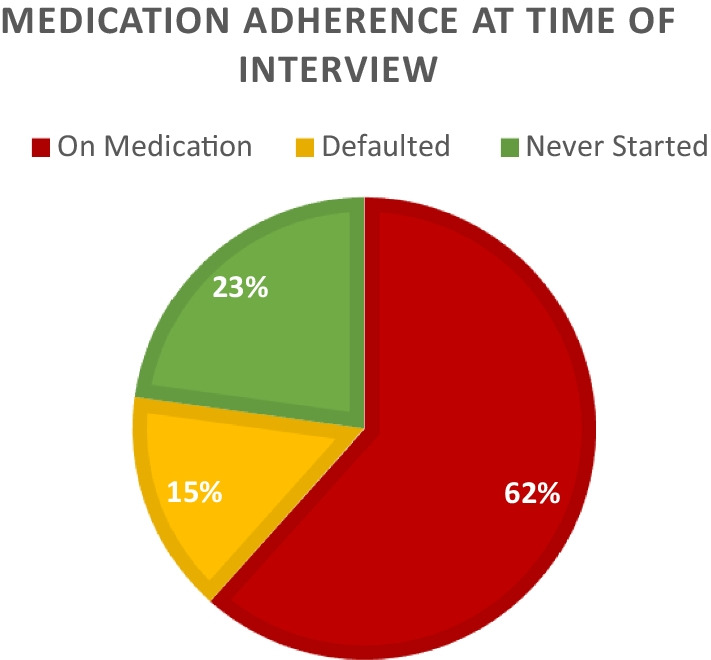
On Medication: Defaulted, Never Started

In Malaysia, HIV is a widespread public health concern and [2, 17, 50] POC are not only vulnerable to contracting HIV due to their precarious circumstances in the wake of conflict [20], but face challenges in obtaining and maintaining care. Malaysia is among the many countries with results-based evidence of Highly Active Anti-Retroviral Therapy (HAART) medication regimens significantly reducing HIV related mortality/morbidity, and increasing life expectancy; however, adherence to these regimens is an important part of their efficacy [16, 20]. While registered HIV positive POC under the protection of UNHCR Malaysia (Fig. 2) are entitled to life-saving HIV related healthcare services, Chesney [8] argues that the difficulties of adhering to daily medication regimens are only made worse by the precarious nature of living as a refugee/asylum-seeker in Malaysia. Only 62% of UNHCR Malaysia’s HIV positive POC population were on ART and 82% of those who were on ART adhered to medication (Fig. 3) (UNHCR [47].

### Interview design

**Fig. 4:**
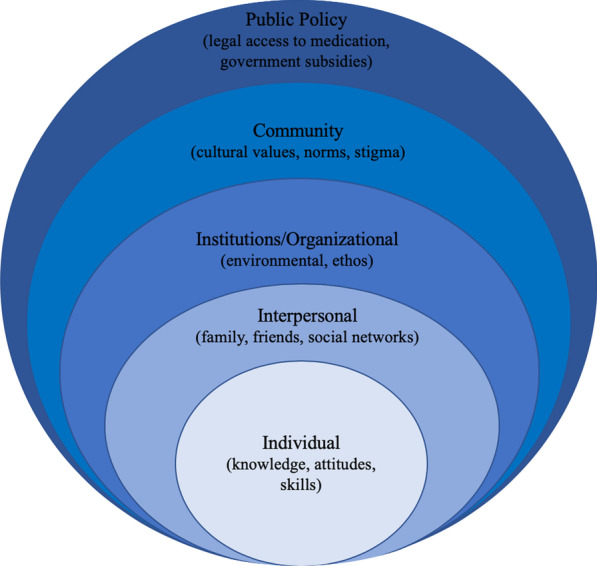
Socio-Ecological Model as applied to HIV adherence in the Malaysia refugee context [23]

Semi-structured, in-depth individual interviews were carried out with HIV positive POCs to gather their perspectives on barriers and facilitators of HIV medication adherence in the setting of Kuala Lumpur, Malaysia [36]. A guiding interview protocol was designed based on the socio-ecological model (SEM) (Fig. 4), which recognises the impact of social and structural determinants on behaviour and health [23]. The five nested, hierarchical levels of the SEM—individual, interpersonal, community, organizational and public policy—cover multifaceted and interrelated layers that can affect HIV medical adherence. This study pays particular attention to the lower four layers,the public policy level is covered in a separate paper [30].

Key interview topics were covered using an interview guide (“Appendix 3”) which combined the use of open-ended and probing questions to gather relevant information and focus participant responses. Interview topics included refugee/asylum-seeker diagnosis experience, experiences of HAART, challenges faced when trying to adhere to medication, perceived facilitators of HAART medication adherence, and potential solutions to improve future adherence. Participants were also asked a series of questions relating to their refugee/asylum seeker status and socio-demographic characteristics. Finally, perspectives on stakeholder (UNHCR, government, NGO’s, pharmacies) interventions to help decrease barriers and increase adherence were explored. Interviewees were asked to consider how factors on all levels (individual, interpersonal, organizational, community, public policy) impacted adherence [5]. This approach was adopted to encourage a more wholistic understanding of HIV medication adherence.

### Participant recruitment and consent

**Fig. 5:**
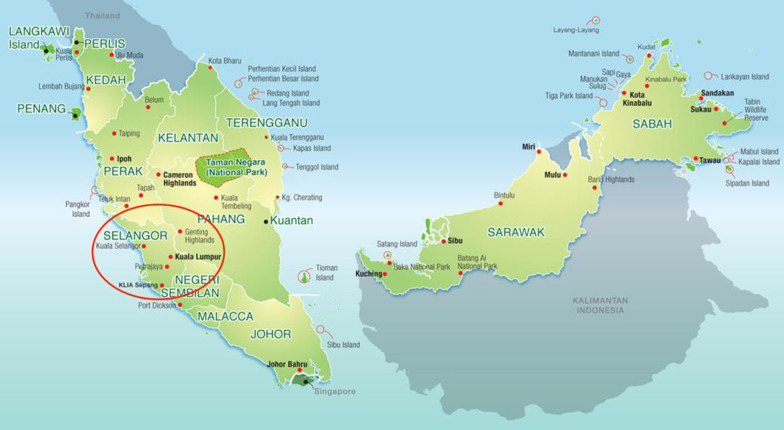
Map of Malaysia with Selangor and Kuala Lumpur Highlighted

As we wished to learn more about the experiences that impacted adherence to medication among HIV positive POCs, we recruited participants from POCs registered with UNHCR Malaysia using the following eligibility criteria: (1) registered and recognized as a refugee or asylum-seeker with UNHCR Malaysia; (2) diagnosed as HIV positive; (3) reside in Selangor or Kuala Lumpur; (4) contactable via phone number; (5) taking medication for minimum 6 months. Participation was restricted to Selangor or Kuala Lumpur (Fig. 5) and to POC who were contactable via telephone for logistical reasons. Eligibility criterion number 5, taking medication for minimum 6 months, was chosen so that POCs would have at least 6 months of experience accessing medication and HIV-related healthcare services. Past UNHCR Malaysia data showed that 6 months was the average time for people to settle on a medication that worked in terms of side effects, finances, and transportation to pharmacies (UNHCR [47].

**Fig. 6:**
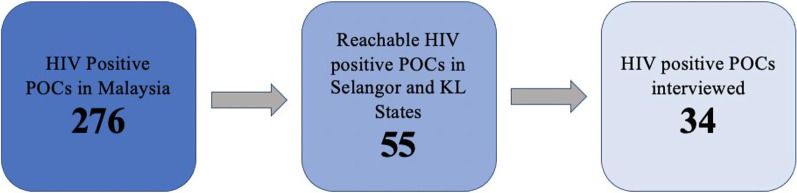
Participant information

Of the 276 POC with HIV registered with the UNHCR in Malaysia, 205 reside in Klang Valley. Of those in the area, 142 individuals were on ART and shortlisted for participation (Fig. 6). The first author and one of three research assistants called each potential participant with the help of an interpreter and, following a participant information sheet (“Appendix 2”), explained the study in full. Specifically, the interpreter translated the author’s words into a language the POC could understand and then again translated the POC’s words back into English for the author to understand. Potential participants were assured that their decision to participate would have no effect on their processing or resettlement status at UNHCR. Interpreters read the full consent form in this initial phone call and explained that participants would either sign the form or, if unable to write, give verbal consent preceding interviews. Consent forms outlined the confidentiality and anonymity on behalf of UNHCR and all researchers involved, as well as the participant’s rights, and contact information that could be used if any questions and/or concerns arose. Consent for audio recording was included in the form as an additional yes/no question. All participants had to give consent before being interviewed. Furthermore, children below 18 that were included in the study had a parent or guardian consent and speak on their behalf. Consent explanations, signing and interviews all took place with children present; however, during interviews, parents alone participated.

All participants were given a RM30 ~ CAD $10 transportation allowance and a small food bag filled with non-perishable items. Preceding the interviews, participants were not made aware that they were receiving any incentives in an attempt to maintain the voluntary nature of participation. REB approval was obtained by Simon Fraser University (REB Number 20190452).

POCs tend to change contact details frequently given their use of prepaid phone cards. Therefore, only 55 POCs were reachable at the time, resulting in 34 individuals interviewed. The discrepancy between those we attempted to contact and those interviewed can be attributed to incorrect/non-updated phone numbers (15), inability to take time off work (4), and health concerns (2) that interfered with participation.

### Data collection

Semi-structured, individual, in-depth interviews were conducted with registered POCs living with HIV from June to July 2019 by the research team—first author or one of three research assistants (two females and one male). The interviews took place in private interview rooms within the UNHCR complex to ensure privacy, familiarity and convenience for participants involved. Thirty-two interviews were completed in person. Two were conducted over the phone as this was more convenient for the participant.

In the interview room there was one interviewer and one interviewee. When needed, one interpreter was utilised in each interview to translate the words of the interviewer and interviewee; they were strictly there to facilitate translation. Interpreters were employed and trained by UNHCR. Each interpreter spoke fluent English as well as their mother tongue– Burmese, Tedim, Rohingya, or Arabic. In an attempt to standardise protocol, interpreters were briefed before each interview, including the circumstances of the POC about to be interviewed, as well as key medical words to ensure they knew how to appropriately translate. Twenty-nine out of the 34 interviews required the assistance of an interpreter.

On average, the interviews lasted 50 min (ranging from 24 to 91 min). Observer details were captured through interviewer journaling directly following the interview. Details included information about the setting of the interview, the time of the interview, observations regarding the interviewee (notes to give insight to body language, and facial expressions), and additional issues or questions that needed to be highlighted to come back to when undertaking the analysis. All interviews were audio-recorded with interviewee consent.

### Data analysis

Following common practices in qualitative research that emphasize the iterative nature of grounded research, data collection and preliminary analysis occurred simultaneously [27]. This allowed the research team to identify new and important perspectives that might have surfaced during interviews and could be used to improve the interview guide. Additionally, the large number of POCs living with HIV in precarious circumstances warranted the need for immediate debriefs. We expected a certain number of POCs to bring up problems that could be fixed with simple but prompt interventions (e.g. communicating with hospitals/pharmacies to secure a single medication pick up time to avoid numerous trips for varying medications). Initially the research team, including first author and all three research assistants, gathered after four interviews for an audio-recorded debrief session in order to identify any striking themes in the data that related to the research focus. Indeed, general themes were identified about linkages between barriers/facilitators and adherence to HIV medication. During subsequent debrief sessions, new data from audio-recordings were compared to existing themes to either strengthen, dispute, or expand upon them.

As interviews were audio recorded according to consent of participants, recordings were transcribed post-interview. The first, second and third authors divided transcriptions (15, 15, and 4 respectively) to read through, double check against original recordings and interviewer notes to ensure accuracy, and further highlight themes within the lower 4 nested levels of the SEM in relation to the primary focus. Through extensive discussion, themes were cross-referenced with other transcription highlights and with key ideas from audio-recording debrief sessions. Through this process, differences were resolved, and common themes emerged. Throughout the highlighting and audio-recording analyses, the individual and interpersonal levels of the SEM were kept in mind to ensure a comprehensive, contextualised understanding of emerging themes.

## Results

### Barriers

#### Stigma

Stigma surrounding HIV was found to be a significant reason for non-adherence to HIV medication. We follow Goffman and define *stigma* as a discrediting attribute that reduces the status of someone in the eyes of their society or community [13]. Stigma can greatly affect the mental, physical, and spiritual wellbeing of individuals who are living with HIV, by impacting aspects of interpersonal relationships with family, friends and community members [1, 6]. All 34 participants mentioned that stigma surrounding an HIV diagnoses had affected their relationships, either directly by exclusion or through more subtle means. It seemed to manifest in many levels of the SEM,specifically, the interpersonal and community level where relationships were impacted. Stigma had also been internalised at the individual level to create feelings of self-doubt, insecurity, and negative self-image.

In the circumstances of POCs’ lives, where social networks are a fundamental resource for survival, many avoided telling loved ones about their status for fear of being of judged or ostracized from their communities. Mr. Ahmad^2^ expressed* “I am bothered by other people’s judgement, and the way they look at me when they find out. It has affected all my relationships”.* As Mr. Ahmad pointed out, the potential negative impact on relationships can be a major deterrent from disclosing one’s HIV status, particularly because it can damage needed social networks.

Participants who had not told friends or family of their positive HIV status were avoidant of taking medication in front of others in order to avoid disclosure and subsequent stigma. For example, Ms. Nur explained “*I used to miss pills many times… because people were with me. The guy with me did not know my HIV status. I worry what people think about me if they know about my status while I am taking pills in front of them.”* As indicated, her worry over what others might think if they saw her taking pills kept her from adhering to medication regularly; she would rather miss a pill than risk other people learning about her medication. This sentiment was not uncommon among participants.

More subtle manifestations of stigmatization surrounding those who disclosed their HIV positive status were also found amongst the study population. Men of the Rohingya population in particular found it difficult to find informal jobs if they disclosed their HIV status to potential employers. As many of the POCs work in construction settings and have reported unsafe or risky working conditions, getting injured or cut is not an uncommon phenomenon (UNHCR [47]. As a safety precaution to preventing transmission, these POCs felt morally obligated to disclose their HIV positive status to employers. Yet, participants reported that disclosure to employers, both potential or current, often resulted in POCs not being hired or losing their job. The stigmatization surrounding HIV manifested as joblessness in many circumstances. Joblessness severely impacted medication adherence in situations where UNHCR was not supporting the cost of medication, as financial constraints were also found as a significant contributing factor to non-adherence.

#### Lack of knowledge

Numerous POCs interviewed felt unaware or uneducated with regards to HIV and their diagnosis, even after consultations with healthcare professionals. Unfortunately, among those that were interviewed, many—particularly Rohingya women—felt that during their initial diagnosis they were not fully briefed on the implications of living with HIV. Ms. Amina told us, *“he (the doctor) told me I had some sort of disease and I that needed to take this medication – that’s it.”* Comprehensive understanding of HIV-specific information, as facilitated by a healthcare professional, has been linked to increased adherence to HIV medication [32]. Evidence suggests that understanding all relevant aspects of an HIV diagnosis can greatly improve medication adherence and decrease risk taking behaviours [11, 54], 32. Yet the lack of information given to Ms. Amina was a common theme that belied a poor understanding of the reasoning behind as well as importance of medication adherence.

Another important theme often raised by the Rohingya population was the sentiment that they were the only ones living with HIV. Several interviewees said variants of Mr. Muhammad’s statement *“…I am the only person with this disease”.* The underestimation of prevalence of HIV among the POC population registered with UNHCR Malaysia augmented interviewees’ feelings of isolation and ostracization within POC communities. The research team members who conducted interviews commonly assured interviewees with UNHCR verified data on prevalence statistics, which both surprised and reassured many. An unintended outcome of this reassurance was that many POCs were more inclined to acknowledge their interest in starting a community-support group and disclosing their status to other POCs living with HIV. As mentioned by Mr. Amir “*I don’t have anyone to check up on me. I want to start a community of people like me so that we can talk, share what we know about [medication] side effects, help others cope mentally, and ask ‘how are you’”*, his interest in connecting with other HIV positive POCs was prompted by his experience with unknown medication side effects regarding what was normal and what warranted medical attention.

#### Language barriers

As discussed in the literature review, a major obstacle to medication adherence was the frequency of misunderstandings between healthcare professionals and POCs living with HIV due to language barriers. Hospitals do not have interpreter staff to cover the need of various POC languages, as Mr. Akmal informed us “*there was no interpreter for me when they told me. It was hard to understand what they were saying”*. Understanding the importance of medication adherence and implications of defaulting treatment was made difficult for Mr. Akmal and many others due to the hinderances in communication. With no way of confidently communicating with healthcare professionals, POCs also found it difficult to ask questions and clarify any points of confusion.

Female POCs expressed concern in feeling like they had to bring their husbands to the pharmacy and hospital whenever appointments were made because their husbands were the only ones who spoke the local language (Malay). Despite the importance of communication, women sometimes reported having to go an appointment or the pharmacy alone. In many circumstances, interviewees reported that husbands were the sole breadwinners and would have to take unpaid time off work in order to accompany their wives. As reported by Ms. Alia, “*My husband has to pick up medication for me and my son, otherwise can’t communicate. He has to take day off to go because it’s far from home, but we have debt to pay.”* Decreases in income due to time off work for hospital or pharmacy appointments can affect a family’s financial solvency, including their ability to afford HIV medication. Ultimately, language barriers frequently put interviewees in the difficult position of choosing between their ability to communicate with providers and their ability to afford medication. Both of these situations present barriers to medication adherence.

### Facilitators

#### Interpersonal relationships

Interpersonal relationships were most frequently expressed by POC’s as a facilitator to HIV medication adherence. Those who had experiences of acceptance by friends, family and community members after disclosing their status often felt more confident and encouraged to adhere to medication regimens. Indeed, disclosure of status was identified as an essential component to receiving social support. The following quote where Ms. Farah comments on her husband’s support illustrates how interpersonal relationships can be significant to dictating the experience of diagnosis and treatment: “*It’s made us closer. He understands. He takes time off work to drive me to appointments and helps me stay healthy”.* Ms. Farah’s experience showcases the immense impact support systems on adhering to medication.

Having support was also found to be an important indicator of perceived stigma surrounding a positive HIV diagnosis. Support from loved ones in the community decreased feelings of isolation, helped HIV positive POCs feel safe, less discriminated against, and more motivated to adhere to medication regimens. Mr. Amir outlined the impact an accepting community has had on his life, “*members of the community come with me to collect my medication, because of the language barrier. Many have even done research on their own to learn [about HIV]”*. As indicted by Mr. Amir, having communities, friends, or family that directly influence adherence in a positive way (transportation, knowledge, financial, reassurance) has been seen to improve adherence.

#### Improved health

Many POCs expressed motivation to adhere to HIV treatment regimens after experiencing the positive health effects of taking medication consistently. Ms. Hana disclosed, *“I always remember to take my medication because it makes me feel better, I no longer have skin wounds or fevers”*—not an uncommon sentiment among the participants. Ms. Hana’s experience reflects the medical knowledge that taking medication or taking it as prescribed is important for the everyday health of people living with HIV; when not controlled with medication, HIV can multiply, making HIV-related illness and disability a real concern [37]. Interviewees reported feeling extraordinary sickness if not on medication, leading to a lack of energy and an inability to participate in daily activities. The possibility of a higher quality of life was a strong motivator to either start taking medication or to comply with treatment regimens. As indicated by Ms. Afra, *“There is no cure – my life stopped moving. I was dizzy, numb, and couldn’t eat. But now that I take medication, I feel fine to live a normal life”.* Notably many interviewees also reported medication side effects difficult to endure. Nevertheless, medication tended to be associated with overall feelings of improved health, which seemed to give POCs hope for a healthy, normal life.

#### Patient-physician relationship

Without exception, POCs that felt supported and comfortable with the doctor or counsellor during diagnosis were the ones who also reported feeling confident managing their disease moving forward. Ms. Hanan disclosed, *“they had a counsellor come and talk to me after the doctor. She explained what HIV was. I was comfortable with the counsellor and understood what she was saying. Even though I was sad, I felt prepared to take medication after leaving the hospital”.* While Ms. Hanan now needed to make certain lifestyle changes, she felt confident moving forward with regard to medication. In this, understanding the implications of not taking medication, or inconsistently taking medication became an important theme that came up, specifically for POCs that had positive healthcare worker-patient relationships. Even though few POCs reported being offered counselling or having good communication with their doctor during their diagnosis conversation, those that were reported feeling better prepared to come to terms with their diagnosis, ask any outstanding questions, educate themselves regarding HIV, and understand the importance of medication adherence.

Language was the second major facilitator of adherence—within the context of patient-physician relationships—that POCs brought up. Effective communication efforts are an essential part of trusting relationships. As language is an important influencing factor of communication, ideal circumstances would see healthcare professionals speaking the same language as their patients and carrying an understanding of patient’s culture. Certain POCs noted having positive experiences with healthcare professionals when interpreters were present, for example Mr. Hasan mentioned, *“he told me in a private room with an interpreter. I was very glad to speak my language. [The doctor] was friendly, he said don’t worry. He wrote a letter to the UN so I could get registered. He tried to help and support me”.* Having a doctor willing to ensure understandability impacted Mr. Hasan’s outlook on medication adherence. However, as Malaysia has a diverse refugee/asylum-seeker demographic, this is not always possible. Overall, POCs that were fortunate enough to have an interpreter present during their diagnosis conversation noted feeling significantly more confident in understanding how to adhere and why to adhere to medication.

## Discussion

Our in-depth qualitative inquiry attending to the complexity of POCs lives generated important insights into the issue of HIV medication adherence among POCs living without regular status. Consistent with studies completed in settlement camps [24, 29] or resettlement destinations [51, 52], our study in the urban and irregular setting of Malaysia found primary barriers to adherence can be categorized as stigma, lack of knowledge of HIV, and language differences, and facilitators as interpersonal relationships, improved health, and positive physician–patient relationships. The categories of perceptual facilitators and barriers fit within the SEM’s hierarchal framework, which explains the multidimensional and interactive influence of personal and environmental factors. The lower four nested levelsindividual, interpersonal, community, and organizations—are particularly relevant and demonstrate the need for interventions that can address the complex interplay between the lower four SEM levels.

As noted above, interpersonal relationships were most frequently expressed by POC’s as a facilitator to HIV medication adherence. At the interpersonal level, social networks and social support systems can positively influence individual behaviours [14]. Supported by the literature, informational, emotional, management, and instrumental support have all been shown to enable forced migrants in different settings to adhere to HIV medication [31, 51, 52]. By having differing types of support, refugees were able to better access care, cope with their diagnosis, and improve adherence [31, 51, 52]. POCs living in Malaysia were no exception,they found social support imperative in dictating their experiences of diagnosis, treatment, and perceived stigma. During interviews, a gap in support was noted by many POCs who expressed their feelings of isolation, loneliness, and ostracization. Having a support network of people who are aware of one’s HIV status can have an immensely positive impact on adherence to medication [1, 6].

With this in mind, fostering social support could potentially be used as a foundational ideology for community-based HIV support groups. The lack of strong social networks indicates the need and potential benefit of having a community support group that could meet regularly and help each other through the numerous challenges expressed. Foreseen benefits are consulting each other about coping mechanisms, medication and common side effects, helping one another find jobs that have non-discriminatory employers or pose less of a transmission risk, helping pick up medication from clinics, giving each other a platform to discuss innovative solutions to some of the challenges of adherence, as well as knowledge transmission of general information regarding HIV. Supported by interventions suggested by Jobarteh et al. [19] in non-refugee populations, peer support can help overcome the barrier of far distances to clinics by creating groups that rotate to help each other pick up medication. From the SEM lens, a support group could greatly increase a sense of belonging within a community of people with similar experiences and struggles and give an opportunity for POCs to come together in solidarity with one another.

Patient-physician relationships are another major factor influencing HIV medication adherence, as relayed by POCs. The interplay of this particular interpersonal relationship with the individual, community and organizational levels of the SEM demonstrate its complexity and need for nuanced analysis. Consistent with the literature, POC’s felt that strong patient-physician relationships were important for feeling confident managing their disease, understanding its implications, and feeling prepared to take medication consistently moving forward. On the other hand, studies showed that individuals with weak patient-physician relationships were less exposed to HIV information, which led to poor understanding of dosing regimens and HIV medication [7, 12]. The connection between patient-physician relationships and HIV knowledge is a particularly important point to emphasize. This interpersonal relationship can heavily influence the individual SEM level regarding knowledge, attitude, behaviour, expectations, and perceived stigma of POCs. It can also lead to a positive or negative relationship with service delivery and institutional or organizational support systems (such as the health care system).

Given that many POCs found language differences a significant barrier to positive patient-physician relationships, consequently leading to lack of HIV knowledge, educational programming could be a way to fill the informational gap. Education related to antiretroviral drugs and risk-taking behaviour has been shown to increase adherence and decrease transmission in both POC and non-POC populations [10, 11, 54]. Evidence from individual in-depth interviews with POCs indicated the need for HIV-related education. A multitude of factors could be contributing to the lack of information given to patients,however, it is important for people living with HIV to know fundamental information about their diagnosis in order to understand the importance of adherence to medication*.* When asked, individual counselling was most preferred among the populations, giving an opportunity to thoroughly assess understanding and encourage questions throughout. Based on our conversations with participants, who had already consulted healthcare professionals, POCs need continuing access to foundational health education regarding the following topics:

- What HIV is
- How many other people have it (statistics)
- Common HIV symptoms
- Precautions to take against transmission
- How medication works
- What the implications of not taking medication are
- Normal and uncommon medication side effects

As mentioned above, the other theme often raised was that POCs thought they were the only ones living with HIV. Keeping in mind that this may stem from a lack of awareness surrounding HIV, the need for education on prevalence statistics is even more pertinent. As opposed to the individual educational counselling, this knowledge translation could be done in a group setting—perhaps during the community support groups—to showcase a concrete manifestation of those statistics. Having a support network that prioritizes education could be an impactful way to decrease feelings of isolation and ostracization born from awareness gaps, ultimately increasing adherence to HIV medication.

Overall, the importance of providing refugees with HAART is clear given how much we know from the evidence presented above as well as prior work, regarding how best to support them in sustaining viral suppression [26].

### Strengths and limitations

The findings of this study add to the literature by situating the barriers and facilitators that influence adherence to HIV medication among urban, POCs with irregular status within the SEM framework. Capturing the viewpoint of POCs themselves was a unique perspective that has added to the body of literature of refugee access to HIV medication. The themes of social support, interpreter-facilitated communication, and education are recommendations that POC felt were important in helping to improve their adherence. In this, the use of semi-structured in-depth interviews enabled exploration of topics and themes beyond the breadth of the interview guide.

There were also several limitations to this study. Firstly, only POCs from Kuala Lumpur and Selangor states were called and asked to participate. Both highly populated and close to the UNHCR office and main pharmacy, the relatively easy access to HIV-related healthcare services may limit the representativeness, especially for rural living individuals. Secondly, availability of interpreters could have limited the fidelity of the perspectives we sought. On rare occasions, language-specific interpreters would not be available (due to high demand within other UNHCR departments) during our scheduled interview time slots. In these cases, the interviews had to occur in either English or Malay, which may not have been ideal for understandability or comprehensive information sharing. Additionally, interpreter gender could have been an impediment to honest communication with POCs of a differing gender (i.e., male interpreter translating for a female POC). Lastly, as this research study was conducted as an independent study commissioned by UNHCR Malaysia to inform and strengthen programmatic interventions, participants may have been hesitant to fully disclose information that they felt might negatively impact their relationship with the UNHCR.

## Conclusion

This is the first study we know of that uses the SEM to examine qualitative evidence regarding experiences of refugees/asylum-seekers who live irregularly in an urban context, despite how common this situation is for people who must flee their home countries. Study results showed that participants’ experiences of barriers (stigma, language, and knowledge) and facilitators (support, patient-physician relationships, improved health), were complex, contextual, and multifaceted in nature. Medication adherence was found to be influenced at the intersection of various social factors such as gender and ethnicity. We concluded that in order for interventions to effectively improve adherence to HIV medication, they must consider the social influences at all levels of the SEM and most importantly, take into account the perspectives and wants of the POC community themselves. Overall, this novel focus led to many suggestions that could help improve HIV medication adherence among refugees and asylum seekers, particularly those in low-income settings.

## Data Availability

Raw data is not available to the public as a matter of confidentiality.

## Appendix 1

See Figs. 1, 2, 3, 4, 5 and 6.

## Footnotes

1 Instrumental support can be defined as assistance in the form of actions that help people meet their needs (ex. driving, babysitting, cooking) [9]

2 Pseudonyms have been created to give voice to the individual participants and protect their identity.
